# Preventing Falls and Malnutrition among Older Adults in Municipal Residential Care in Sweden: A Registry Study

**DOI:** 10.1177/23779608211026161

**Published:** 2021-07-07

**Authors:** Annelie K. Gusdal, Rose-Marie Johansson-Pajala, Marina Arkkukangas, Anna Ekholm, Viktoria Zander

**Affiliations:** 1 School of Health, Care and Social Welfare, Mälardalen University, Eskilstuna/Västerås, Sweden; 2 Research and Development in Sörmland, Eskilstuna, Sweden

**Keywords:** fall, malnutrition, older adults, prevention, quality registry

## Abstract

**Introduction:**

Older adults in municipal residential care are among the most vulnerable and in need of most care. The prevalence of negative events, such as falls and malnutrition, is increased among these older adults. The need for strategies to prevent falls and malnutrition is emphasized in guidelines and systematic, individualized risk assessments are prerequisites for adequate interventions.

**Objectives:**

The overall purpose of this study was to investigate the assessed risks of, and risk factors for, falling and malnutrition and the correlations between these assessed risks among older women and men in residential care. Further, the purpose was to investigate the consistency between planned and performed interventions among women and men assessed as at risk.

**Methods:**

A cross-sectional registry study based on risk assessment data in the Swedish national quality registry, Senior Alert. Altogether, 5,919 older adults ≥65 in nursing homes and dementia care units in 19 municipalities in Sweden were included.

**Results:**

Of the older adults, 77% were at risk of falls, and 59% were at risk of malnutrition. The most prevalent risk factors for falls were previous falls and not being cognitively oriented; and for malnutrition were having mild or severe dementia or depression. A significant positive correlation between the risk of falling and the risk of malnutrition was found. Less than half of the planned interventions for falls and malnutrition were performed. Care staff’s least common interventions to prevent falls were balance, muscular function, and strength training, which contrasts with the recommendations; interventions to prevent malnutrition were only partially adhering to recommendations.

**Conclusions:**

This cross-sectional registry study points towards the importance of using an evidence-based approach, based on adherence to recommended guidelines, in the prevention of falling and malnutrition. Further, the implementation of clinical practice guidelines is needed, which requires educational training for care staff and supportive leadership.

## Background

The older population is increasing worldwide. Currently, 125 million adults are aged 80 or older, and by 2050 there will be 434 million adults in this age group (World Health Organization [WHO], 2017a). Despite recent positive findings that the older population is more active and healthier than ever before (Falk et al., 2014), aging is still associated with declining physiological functions. In Sweden, approximately 536,000 adults are aged 80 or older, and 80,000 adults live in municipal residential care permanently (Statistiska centralbyrån, 2020). These 80,000 are particularly vulnerable, being the oldest, frailest, and generally in need of the most care (Lagergren, 2002). Also, the prevalence of negative events, such as falls and malnutrition, increases among these older adults (Ernsth Bravell et al., 2011; Fried et al., 2004; Kojima, 2015; Verlaan et al., 2017). Falls and malnutrition are interrelated and considered as frequent and serious events for older adults (Meijers et al., 2012; Neyens et al., 2013).

Malnutrition is often defined as undernourishment due to insufficient dietary intake, poor appetite, muscle wasting and weight loss (Chen et al., 2001) and is a predictor for the onset of morbidity and mortality among older adults (Abizanda et al., 2016; Borgström Bolmsjö et al., 2015; Norman et al., 2008). The prevalence of malnutrition is well documented. In a systematic review and meta-analysis of international research, the prevalence and/or risk of malnutrition in nursing homes and long-term care was reported to be 66% (Cereda et al., 2016). In two Swedish studies conducted in nursing homes, the prevalence and/or risk of malnutrition was estimated to be 58% (Borgström Bolmsjö et al., 2015; Lannering et al., 2016), results that correspond to data from the Swedish National Board of Health and Welfare (Socialstyrelsen [SoS], 2020b). There are multiple grounds for malnutrition and weight loss among older adults in nursing homes, such as old age, dementia, depression, poor oral intake, swallowing issues, eating/chewing dependency, immobility (Tamura et al., 2013) and low staffing levels (Woo et al., 2005). Undetected and untreated malnutrition reduces physical and mental functions and impairs recovery, and it can contribute to a loss of autonomy and reduced quality of life. However, malnutrition is preventable and mostly reversible with early adequate nutritional therapy. It often remains undetected due to lack of awareness, knowledge, and clinical protocols to detect and treat. A systematic and standardized approach to identifying malnutrition is needed, and that is where nutritional screening tools play an important role (Artaza-Artabe et al., 2016; Bauer et al., 2008).

There is a significant relationship between malnutrition, sarcopenia, and the risk of falling; therefore, malnutrition and sarcopenia must be addressed to reduce that risk (Meijers et al., 2012; Neyens et al., 2013; Sieber, 2019). Sarcopenia is defined as an increased loss of muscle mass and muscle function while aging (Santilli et al., 2014), and is associated with a decrease in mobility, balance and muscle strength and is thus a predisposing factor related to falling. A sarcopenia diagnosis is not part of the standard diagnostic and therapeutic repertoire of geriatric medicine and is therefore often overlooked (Sieber, 2019). The number of older adults who have experienced a fall is reported to be over 30% for adults 65 and older, and the incidence rates of falls among older adults in municipal residential care are even higher due to their increased frailty (Cameron et al., 2018). Falls are the second leading cause of accidental or unintentional injury deaths worldwide (WHO, 2018), and in Sweden 90% of all injuries among adults 80 and older are caused by a fall (SoS, 2018). Due to the demographic trend, the number of fatal fall accidents can be expected to increase dramatically in the coming decades (Myndigheten för Samhällsskydd och Beredskap, 2014). The risk factors for falls and fall-related injuries are well documented. The most dominant risk factors besides age, gender, and psychological functions such as fear of falling and low self-efficacy are reduced muscle strength, reduced balance, and a high number of diseases (Arkkukangas et al., 2020; Damián et al., 2013; Deandrea et al., 2010).

The consequences of falls and malnutrition are extensive and include morbidity, mortality, and increased healthcare costs (Neyens et al., 2013; Norman et al., 2008) and the need for strategies to prevent malnutrition and falls is emphasized in guidelines and research literature (Sherrington et al., 2020; WHO, 2017b). Early detection of risk of falling and malnutrition, planning, and performing adequate intervention are crucial in older adults to prevent serious events. Thus, systematic and individualized assessments of the risk of falling and malnutrition can guide the decisions regarding suitable interventions (Artaza-Artabe et al., 2016; Bauer et al., 2008), together with relevant decision support systems (Fossum et al., 2011). However, previous research conclude that older adults assessed to be of risk of falling and/or malnutrition do not receive planned and performed interventions to the expected degree and there is a mismatch between assessed risk factors, and planned and performed interventions (Backlund et al., 2018; Witt et al., 2018). It is thus critical to gain further knowledge about to which extent guidelines and evidence-based practice are used when planning and performing interventions for older adults living in residential care. The overall purpose of this study was to investigate the assessed risks of, and risk factors for, falling and malnutrition and the correlations between these assessed risks among older women and men in residential care. Further, the purpose was to investigate the consistency between planned and performed interventions among women and men assessed as at risk.

## Methods

### Design

This is a cross-sectional registry study based on data from the Swedish national quality registry, Senior Alert (SA). SA is accessed via the internet and is a tool to support a standardized, structured, and systematic preventive care process for older adults. An individual may be risk assessed using SA upon receiving a care contact and information about the registry from the healthcare provider. Follow-up risk assessments are performed once-twice yearly, or more often if the individual experiences a change or deterioration in health status. Its aims are to improve health outcomes, facilitate systematic follow-ups and reduce costs for society (Edvinsson et al., 2015; SA, 2020). SA is one of more than 100 national quality registries in Sweden, and its use has increased rapidly since it was established in 2008. In 2019, 277 of Sweden’s 290 municipalities and 13 of Sweden’s 21 regions actively registered with SA (personal communication with SA’s registry holder Erica Löwhagen, 2020-09-09). SA is unique, as it is not disease-specific; instead, it focuses on a structured preventive care model built on nursing variables to improve the quality of care. The preventive care model includes four steps - risk assessment using valid and reliable assessment instruments, analysis of causes when risk exists, planned, and performed actions and evaluation. SA covers five risk areas - falls, malnutrition, pressure ulcers, oral health, and bladder dysfunction. The risk areas are related, and it is mandatory to assess risks in three of the risk areas (falls, malnutrition, pressure ulcers) in adults ≥ 65 (Edvinsson et al., 2015).

### Sample and Setting

The sample consisted of 5,919 older adults ≥ 65, both women and men. There were 10,541 risk assessments performed for these older adults and the last risk assessment was used in the present study. Of the 5,919 older adults, 4,850 (82%) lived in nursing homes and 1,069 (18%) lived in dementia care units in 19 municipalities in two counties in the mid-east of Sweden. Other settings, such as hospitals, short-term stays at nursing homes, and home health care were excluded as SA has a lower coverage ratio in these care units.

In 2018 in Sweden, municipal residential care was granted for approximately 111,000 people aged 65 or older, in 2,285 different care units, compared to approximately 283,000 people aged 65 or older who received municipal home care (SoS, 2020c). Of the residential care units, 19% are run by private actors (Vårdföretagarna Almega, 2019). Municipal residential care includes both general elderly care and dementia care. The employees mostly consist of unlicensed care staff, i.e., assistant nurses and care assistants, who amounted to approximately 136,000 in 2017. The licensed care staff, i.e., registered nurses, physiotherapist, and dietitians constitute a smaller part of the employees; and for example, the number of registered nurses (RNs) amounted to approximately 14,000 in 2017 (SoS, 2020c). The RNs have the overall responsibility for the care and have a consultative role in relation to the unlicensed personnel who perform the daily bedside care (Furåker & Nilsson, 2013). The municipalities do not have medical doctors employed, instead the municipalities buy medical services from regionally or privately employed medical doctors.

### Data Collection

The data collected by care staff covered the period between 1 September 2018 and 19 February 2020. The following variables were gathered from SA: county, municipality, type of care unit, age group, biological sex, body mass index (BMI), date of risk assessment, risk assessment based on the Downton Fall Risk Index (DFRI) (Downton, 1993; Rosendahl et al., 2003), risk assessment based on the Mini Nutritional Assessment - Short Form (MNA-SF) (Rubenstein et al., 2001) and planned and performed interventions to prevent falls and malnutrition (SA, 2020).

### Instruments

DFRI is an instrument to assess the risk of falling. It has shown high sensitivity and lower specificity but is still considered to have adequate predictive accuracy among older adults (Rosendahl et al., 2003). Eleven risk factors are grouped into five categories: 1) previously known falls; 2) medication: sedatives/hypnotics/neuroleptics (note: measured as one risk factor), diuretics, antihypertensives, anti-Parkinson’s medication, antidepressants; 3) sensory impairments: impaired vision, impaired hearing, impaired motor skills; 4) cognitive ability: not oriented; and 5) walking ability: impaired walking ability. Each of the 11 risk factors is scored either as a 0 or a 1, yielding a maximum score of 11 points in DFRI. Scores ≥3 indicate a high risk of falling (Downton, 1993; Rosendahl et al., 2003).

MNA-SF is an instrument to assess the risk of malnutrition. Eleven risk factors are grouped into six categories: 1) decrease in food intake in the last 3 months (0–2 points); 2) involuntary weight loss in the last 3 months (0–3 points); 3) mobility (0–2 points); 4) mental stress or acute illness (0 or 2 points); 5) neuropsychological problems (0–2 points); and 6) BMI (0–3 points). The maximum score is 14 points in MNA-SF. Scores of 8–11 points indicate a risk of malnutrition, and scores of 0–7 points indicate malnutrition (Guigoz et al., 1996; Kaiser et al., 2009).

### Data Analyses

Descriptive statistics of demographic data were calculated. Mann–Whitney *U* test and Pearson's chi-square test were used to analyse differences between two subgroups, and independent T-test was used to compare means. Correlations between fall and malnutrition were analysed using Spearman’s rho. Logistic regression analyses were used to analyse malnutrition risk factors as predictors for the risk of falling. Two absurd BMI scores (149 and 161) were replaced using imputation of means. The overall significance level was set at *p* ≤ .05. Statistical analyses were performed using IBM SPSS Statistics version 26.0 for Windows.

### Ethical Approval

The study was approved by the Swedish Ethical Review Authority (Dno. 2019-03767) and the extradition of data was approved by the national registry SA. The data was decoded during the extradition of data and was not altered afterwards. The study conforms to the principles outlined in the Declaration of Helsinki (World Medical Association, 2015).

### Consent to Participate and Consent for Publication

Swedish national quality registries such as SA are regulated by Swedish law in the Patientdatalagen (The Patient Data Law) (SFS, 2008), which stipulates that personal data may not be processed in a quality registry such as SA if the individual is opposed. Prior to registration, the healthcare provider has the legal obligation to inform the individual about the registry and data collection, whereupon the individual gives their informed consent to participate. The individual has the legal right to have personal information removed from the registry at any time (Edvinsson, 2015; World Medical Association, 2015).

## Results

### Description of Study Population

The results are based on 5,919 older adults, of which 4,850 (82%) lived in nursing homes and 1,069 (18%) in dementia care units. A majority were women (*n* = 3,861, 65%), and a majority was in the age group 80-89 years (*n* = 2,450, 41%) with a higher proportion of women (*n* = 1,578, 64%). An even higher proportion of women (*n* = 1,607, 76%) was in the oldest age group ≥90 years (*n* = 2,110, 36%) (Table 1).

**Table 1.** Description of Study Population.

Variables	All *n* = 5,919 (100%)	Women *n* = 3,861 (65%)	Men *n* = 2,058 (35%)	*p*-value^a^
Age group (range 65–106 years)				**<.000**
≤79 years	1,359 (23%)	676 (50%)	683 (50%)	
80–89 years	2,450 (41%)	1,578 (64%)	872 (36%)	
≥90 years	2,110 (36%)	1,607 (76%)	503 (24%)	
Type of care unit				.407
Nursing home	4,850 (82%)	3,152 (65%)	1,698 (35%)	
Dementia care unit	1,069 (18%)	709 (66%)	360 (34%)	

Significant *p*-values in boldface.^a^Pearson’s chi-square test indicating the significance of distribution in age group and type of care unit.

There was a statistically significant difference between women and men in the mean BMI scores. There were no statistically significant differences between women and men in the median scores of DFRI and MNA (Table 2).

**Table 2.** Differences Between Women and Men According to BMI, DFRI and MNA-SF.

Variables	All *n* = 5,919 (100%)	Women *n* = 3,861 (65%)	Men *n* = 2,058 (35%)	*p*-value
BMI, mean (SD)	25.0 (5.3)	24.8 (5.49)	25.2 (4.81)	**.007** ^a^
DFRI, median (min-max) Missing data (*n* = 133, 2%)	4 (0–10)	4 (0–10)	4 (0–9)	.092**^b^**
MNA-SF, median (min-max) Missing data (*n* = 134, 2%)	11 (0–14)	11 (0–14)	11 (1–14)	.204^b^

BMI=body mass index, DFRI= Downton Fall Risk Index, MNA-SF=Mini Nutritional Assessment-Short Form, SD=standard deviation. DFRI - scores ≥3 indicate a high risk of falling. MNA-SF - scores of 8–11 points indicate a risk of malnutrition, and scores of 0–7 points indicate malnutrition.Significant *p*-values in boldface.

^a^Independent t-test.

^b^Mann-Whitney U-test.

### Risk of Falling and Risk Factors for Falling

Among the older adults, 4,563 (77%) were at risk of falling. Of these, 18% (*n* = 819) lived in a dementia care unit and 82% (*n* = 3,744) lived in a nursing home. In the dementia care units, 77% (*n* = 819) of the older adults were at risk of falling, and the corresponding proportion in the nursing homes was 77% (*n* = 3,744). The two most prevalent risk factors for falling were having fallen previously (*n* = 2,914, 49%) and not being cognitively oriented (*n* = 2,988, 51%). Among medications, antihypertensives were most prevalent (*n* = 2,668, 47%). Among older adults with sensory impairments, having impaired vision was the most prevalent risk factor for falling (*n* = 2,312, 61%). Having impaired walking ability was also a risk factor for falling (*n* = 1,770, 30%), as was safe walking ability with or without aides (*n* = 2,624, 44%) (Table 3).

**Table 3.** Risk of Falling and Risk Factors for Falling According to DFRI.

Variables	All *n* = 5,919 (100%)	Women *n* = 3,861 (65%)	Men *n* = 2,058 (35%)	*p*-value^a^
Risk of falling	4,563 (77%)	2,981 (77%)	1,582 (77%)	.769
Previously known falls				
Yes	2,914 (49%)	1,865 (48%)	1,049 (51%)	
Missing data	133 (2%)			
Medications				
No medication	95 (2%)	63 (2%)	32 (2%)	.94
Sedatives/hypnotics/neuroleptics	2,552 (45%)	1,738 (47%)	814 (41%)	**.000**
Diuretics	1,643 (29%)	1,069 (29%)	574 (29%)	.94
Antihypertensives	2,668 (47%)	1,774 (48%)	894 (45%)	**.044**
Anti-Parkinson’s medication	261 (5%)	124 (3%)	137 (7%)	**.000**
Antidepressants	2,373 (42%)	1,605 (43%)	768 (39%)	**.001**
Other medicationMissing data	4,508 (79%)228	2,955 (80%)	1,553 (78%)	.203
Sensory impairments				
Impaired vision	2,312 (61%)	1,536 (64%)	776 (56%)	**.000**
Impaired hearing	1,655 (44%)	1,039 (43%)	616 (44%)	.576
Impaired motor skills	1,716 (45%)	989 (41%)	727 (52%)	**.000**
Missing data	2,123			
Cognitive ability				
Not oriented	2,988 (51%)	1,987 (52%)	1,001 (49%)	.138
Missing data	133			
Walking ability				
Safe with or without aides	2,624 (44%)	1,821 (47%)	803 (39%)	**.000**
Impaired walking ability	1,770 (30%)	1,063 (28%)	707 (34%)	**.000**
No walking ability	1,392 (24%)	886 (23%)	506 (25%)	.156
Missing data	133			

DFRI= Downton Fall Risk Index.Significant *p*-values in boldface.

^a^Pearson’s chi-square test.

### Risk of Malnutrition and Risk Factors for Malnutrition

Among the older adults, 3,465 (59%) were at risk of malnutrition. Of these, 20% (*n* = 697) lived in a dementia care unit and 80% (*n* = 2,768) lived in a nursing home. In the dementia care units, 65% (*n* = 697) of the older adults were at risk of malnutrition, and the corresponding proportion in the nursing homes was 57% (*n* = 2,768). The two most prevalent risk factors for malnutrition were having mild dementia or depression (*n* = 2,530, 44%) or severe dementia or depression (*n* = 1,440, 25%). Other risk factors for malnutrition were having had a recent slight decrease in food intake (*n* = 1,058, 18%), recent involuntary weight loss of 1–3 kg (*n* = 1,085, 21%), being bedridden or wheelchair bound (*n* = 1,468, 25%), or getting out of the bed/wheelchair but not going outside (*n* = 942, 16%). Having had recent mental stress or acute illness was another risk factor for malnutrition (*n* = 1,207, 12%) (Table 4).

**Table 4.** Risk of Malnutrition and Risk Factors for Malnutrition According to MNA-SF.

Variables	All *n* = 5,919 (100%)	Women *n* = 3,861 (65%)	Men *n* = 2,058 (35%)	*p*-value^a^
Risk of malnutrition	3,465 (59%)	2,281 (59%)	1,184 (58%)	.250
Decrease in food intake in the last 3 months				.331
Considerable decrease	291 (5%)	200 (5%)	91 (5%)	
Slight decrease	1,058 (18%)	697 (18%)	361 (18%)	
None	4,436 (77%)	2,872 (76%)	1,564 (78%)	
Missing data	134			
Involuntary weight loss in the last 3 months				**.001**
>3 kg	550 (10%)	324 (9%)	226 (13%)	
1–3 kg	1,085 (21%)	743 (22%)	342 (19%)	
None	3,621 (69%)	2,384 (69%)	1,237 (69%)	
Missing data	663			
Mobility				.079
Bedridden or wheelchair bound	1,468 (25%)	934 (25%)	534 (26%)	
Gets out of bed/wheelchair but	942 (16%)	596 (16%)	346 (17%)	
does not go outside				
Goes outside with or without aides	3,375 (58%)	2,239 (59%)	1,136 (56%)	
Missing data	134			
Mental stress or acute illness in the last 3 months				.098
Yes	1,207 (12%)	762 (20%)	445 (22%)	
No	4,578 (79%)	3,007 (80%)	1,571 (78%)	
Missing data	134			
Neuropsychological problems				.146
Severe dementia or depression	1,440 (25%)	966 (26%)	474 (24%)	
Mild dementia or depression	2,530 (44%)	1,645 (44%)	885 (44%)	
None	1,815 (31%)	1,158 (21%)	657 (33%)	
Missing data	134			

MNA-SF=Mini Nutritional Assessment-Short Form.

Significant *p*-values in boldface.^a^Pearson’s chi-square test.

### Correlations Between Fall and Malnutrition

A total of 49% of the older adults had both a risk of falling and a risk of malnutrition (*p* < .001) and there was a statistically significant positive correlation between the risk of falling and the risk of malnutrition (*rs* = .176, *n* = 5,919, *p* < .001, two-tailed). The logistic regression analyses (Table 5) suggest that all risk factors for malnutrition, except decrease in food intake, were significant predictors for the risk of falling.

**Table 5.** Logistic Regression Analyses of Malnutrition Risk Factors as Predictors for the Risk of Falling, Adjusted for Sex and Age.

	Risk of falling
Malnutrition risk factors	OR (95% CI)
Slight and considerable decrease in food intake in the last 3 months	1.17 (.96–1.42)
Involuntary weight loss in the last 3 months 1–> 3 kg	1.30 (1.10–1.54)*
Mobility	
Gets out of bed/wheelchair but does not go outside	1.92 (1.63–2.27)**
Goes outside with or without aides	4.18 (3.25–5.38)**
Mental stress or acute illness in the last 3 months	2.07 (1.68–2.54)**
Neuropsychological problems	
Mild and severe dementia or depression	2.09 (1.82–2.40)**

*p < .01; **p < .001; OR = Odds Ratio; CI = Confidence Interval.

### Planned and Performed Interventions in Relation to Assessed Risks of Falling and Malnutrition

A total of 4,563 older adults were assessed as being at risk of falling. Of these, 92% (*n* = 4,200) were planned to receive intervention/s; 44% (*n* = 2,005) of the planned interventions were also performed (Table 6). There were statistically significant differences between the care units; in the dementia care units, 81% of the planned interventions were performed, whereas in the nursing homes 74% of the planned interventions were performed (*p* = .002).

**Table 6.** Planned and Performed Interventions in Relation to Assessed Risks of Falling and Malnutrition.

Variables	All *n* = 5,919 (100%)
Planned interventions^a^	Risk of fall, *n* = 4,563 (77%)
Yes	4,200 (92%)
No	49 (1%)
Missing	314 (7%)
Performed interventions^b^	Risk of fall, *n* = 4,563 (77%)
Yes	2,005 (44%)
No	688 (15%)
Missing	1,870 (41%)
Planned interventions^a^	Risk of malnutrition, *n* = 3,*465* (59%)
Yes	3,180 (92%)
No	33 (1%)
Missing	252 (7%)
Performed interventions^b^	Risk of malnutrition, *n* = 3,465 (59%)
Yes	1,543 (45%)
No	547 (16%)
Missing	1,375 (40%)

^a^Missing data for planned interventions (*n* = 797, 13%).

^b^Missing data for performed interventions (*n* = 2,691, 45%).

A total of 3,465 older adults were assessed to be at risk of malnutrition. Of these, 92% (*n* = 3,180) were planned to receive intervention/s; 45% (*n* = 1,543) of the planned interventions were also performed (Table 6). There were statistically significant differences between the care units; in the dementia care units, 81% of the planned interventions were performed, whereas in the nursing homes 72% of the planned interventions were performed (*p* < .000).

There was no statistically significant difference between women and men regarding the planned and performed interventions related to neither the assessed risk of falling nor the assessed risk of malnutrition.

### Types of Performed Interventions

**Figure 1.**
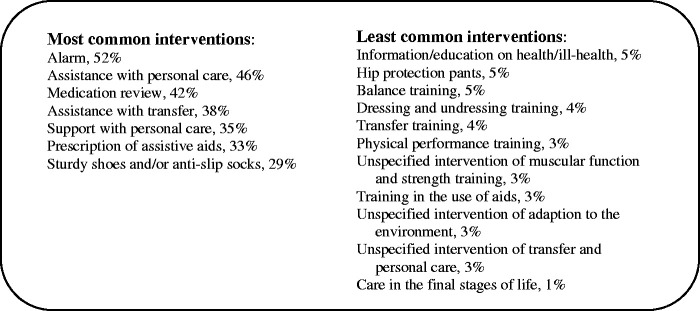
Most and Least Common Performed Interventions Related to Risk of Falling.

Among the older adults who were assessed as being at risk of falling, 2,005 received 24 different interventions with a mean of 3.8 interventions per person. The most common interventions (received by ≥25% of the older adults) and the least common interventions (received by ≤5% of the older adults) are presented in Figure 1.

**Figure 2.**
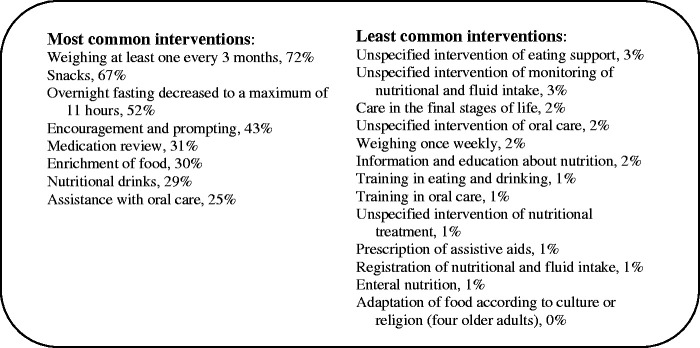
Most and Least Common Performed Interventions Related to Risk of Malnutrition.

Among the older adults who were assessed as being at risk of malnutrition, 1,543 received 26 different interventions with a mean of 4.5 interventions per person. The most common interventions (received by ≥ 25% of the older adults) and the least common interventions (received by ≤ 3% of the older adults) are presented in Figure 2.

## Discussion

The study results showed that a high proportion of the 5,919 older adults were at risk of falling (77%) and malnutrition (59%), which is consistent with the findings of previous registry studies using SA (Backlund et al., 2018; Lannering et al., 2016; Lindmark et al., 2018; Witt et al., 2018). The consistency of these proportions across studies and time may be a response to the steady increase in the number of frail older adults living in nursing homes and dementia care units.

The most prevalent risk factors for falling found in the present study were having impaired vision (*n* = 2,312, 61%), having fallen previously (*n* = 2,914, 49%), not being cognitively oriented (*n* = 2,988, 51%), and using medications, particularly antihypertensives (*n* = 2,668, 47%). These risk factors are in line with previous systematic reviews, which also report wandering, Parkinson’s disease, dizziness, gait impairment and trunk restraint use, and the total number of medications as prevalent risk factors for falling (Deandrea et al., 2013; Kröpelin et al., 2013; Muir et al., 2012).

In the present study, a mean of 3.8 performed interventions per person to prevent falling were registered. Having multiple interventions per person is positive, as combining interventions may have an aggregated potential to reduce the risk of falling among older adults (Cameron et al., 2018; Hopewell et al., 2018). The older adults received medication reviews more frequently than the older adults in, for example, the study by Witt and colleagues (2018). Medication-targeted interventions, such as medication reviews and the adjustment of multiple medications in combination with gradual withdrawal from sedatives, anti-anxiety medication and antidepressants, have an effect on the number of falls (Karlsson et al., 2013). Considering that 47% of the older adults in the present study were prescribed antihypertensives and 45% were prescribed sedatives/hypnotics/neuroleptics, it is important to continue systematic medication reviews. Attention to polypharmacy among older adults and decreasing their use of unnecessary and inappropriate medications are important actions and have been highlighted as a prioritized area for most ill older adults in Sweden (Regeringskansliet, 2014). The number of medications increases with higher age, and in nursing homes up to 74% of the residents regularly consume nine or more medications (Jokanovic et al., 2015). These are unsettling numbers, as consuming more than two medications increases the risk of interactions and side effects.

Unfortunately, the other types of interventions to prevent falling in the present study can only be described as passive. The least common interventions were balance and physical performance training and muscular function and strength training, as also found in the study by Witt et al. (2018). To fail to perform the latter interventions contrasts with recommendations stating that evidence-based exercise programs should be prioritised and include progressive strength, balance and functional exercises for older adults in both ordinary housing (Gillespie et al., 2012; Lord & Close, 2018; Sherrington et al., 2020) and municipal residential care (Lee & Kim, 2017). Exercise programs have been shown to be the most effective in terms of outcome and cost and the only intervention to reduce the number of adults who fall (Karlsson et al., 2013; Lord & Close, 2018).

The most prevalent risk factors for malnutrition found in the present study were having neuropsychological problems (that often involve cognitive impairment), such as mild dementia or depression (*n* = 2,530, 44%) and severe dementia or depression (*n* = 1,440, 25%). This is consistent with the findings of several previous studies (Fávaro-Moreira et al., 2016; Galesi et al., 2013; Roqué et al., 2013). Having mild or severe dementia or depression, with only a small difference between women and men, differs from the findings of Corish and Bardon (2019), who report cognitive impairment as a risk factor to a higher degree for women than men. Corish and Bardon (2019) found that declining health and functional status were predictors for malnutrition among men, factors that were not identified in the present study, as health and functional status are not included as variables in the MNA-SF instrument.

In the present study, a mean of 4.5 performed interventions per person to prevent malnutrition were registered. The types of interventions and the multiple interventions per person are in line with national guidelines to prevent and treat malnutrition (SoS, 2020a). However, the occurrence of one of the most appropriate and recommended interventions to prevent and treat malnutrition, assistance with oral care, was relatively low (25%), and the unspecified intervention of oral care, and training in oral care only accounted for 2% and 1%, respectively, of the performed interventions. Although higher than in the findings of Backlund et al. (2018), these numbers are troublingly low considering the importance of oral health status in older adults with nutritional problems (Holst et al., 2013; Lindmark et al., 2018). Lindmark et al. (2018) found that approximately 41% of the older adults in their study, most of whom were living in nursing homes, had moderate to severe oral health problems. Furthermore, several studies report an association between oral health status and malnutrition (Holst et al., 2013; Lindmark et al., 2018; Sumi et al., 2010; Van Lancker et al., 2012). Oral care alone has been shown to help maintain the nutritional status of older adults (Sumi et al., 2010); therefore, it is important for care staff to detect oral health problems in older adults at an early stage to decrease and/or eliminate eating problems (Westergren et al., 2002). Oral health is assessed with the Revised Oral Assessment Guide (ROAG) included in SA, so presumably interventions involving oral care are registered in ROAG. Unfortunately, oral health is not part of one of the three mandatory risk areas to assess, and therefore oral health status and oral care may still be overlooked.

One of the most common interventions to prevent malnutrition were medication reviews. Although medication usage is not included as a risk factor in MNA-SF, polypharmacy is a risk factor for malnutrition as it indirectly affects nutritional intake (Fávaro-Moreira et al., 2016). Several studies also report that nursing home residents are prescribed potentially inappropriate medications (Storms et al., 2017). In the present study, the intake of sedatives/hypnotics/neuroleptics clearly posed a risk (45%), as these medications can cause anorexia or interfere with the ingestion of food (Norman et al., 2008). Furthermore, mild, or severe dementia or depression were dominating risk factors (the proportion of older adults at risk was also larger in dementia care units). Therefore, it would be expected that the intervention of adapting the environment for individual meal situations would be performed to a greater extent, as it was among the most common interventions in the studies by Backlund et al. (2018) and Johansson et al. (2017).

In the present study, significant correlations were found between the risk of falling and the risk of malnutrition. Except for medication reviews, the performed interventions to prevent older adults from falling did not include interventions related to the older adults’ nutritional status, despite the documented association between an increased risk of falling and malnutrition and the association between malnutrition and impaired activity (Neyens et al., 2013). The effectiveness of interventions to prevent falls is debatable in general and in the present study if nutritional aspects are not considered (Neyens et al., 2013; Westergren et al., 2014; Woo et al., 2005).

Further, the purpose was to investigate the consistency between planned and performed interventions among those assessed as being at risk. The older adults who were at risk of falling received 44% of the planned interventions, and those at risk of malnutrition received 45% of the planned interventions. Planned interventions were performed to a lesser degree in nursing homes than in dementia care units. This loss of over half of the planned interventions and the considerable omission to register performed interventions (i.e., large amount of missing data on performed interventions) raises questions. We have no knowledge of whether the interventions were performed but not registered, or if they were not performed at all. It is essential that care staff are motivated and understand the significance of registering and why the use of quality registries could be used as part of quality improvements and educational interventions for care staff (Edvinsson et al., 2015; Trinks et al., 2018). However, the validity and reliability of DFRI has been questioned based on that large number of residents are assessed as being at risk of falling, i.e., the instrument has low specificity (false positives) (Lannering et al., 2016). It has been argued by care staff in nursing homes that the DFRI neither reflect the reality of the older adults, nor result in better clinical outcomes than the reliance on the care staff’s own clinical judgement (da Costa et al., 2012; Meyer et al., 2009). It is therefore emphasized that in addition to the total risk assessment score of DFRI, each assessed item should be considered separately to be able to tailor interventions according to the older adults’ individual needs (Lannering et al., 2016). Furthermore, the population in municipal residential care has changed over the years and is not comparable to the population at the time when the instruments were developed. This mismatch calls for refinements and psychometric reevaluations of the instruments to confirm their validity and reliability (Isautier et al., 2019; Lannering et al., 2016).

The increasing care burden in institutionalized care must also be considered. This burden is well recognised and debated in society, as is the shortage of care staff and high care staff turnover. Under these circumstances, there is an urgent need to update care staffs’ knowledge of older adults’ nutritional needs (Törmä et al., 2013). According to Woo et al. (2005) and Tamura et al. (2013), inadequate staff levels are associated with malnutrition. The risks related to malnutrition are well documented, as are the various interventions that need to be taken. As it is ultimately the care staff who are responsible for assessing, preventing, and taking action; investing in the education of care staff and securing adequate staffing will most likely have a positive impact on residents’ nutritional status and by extension reduce the incidence of falling.

### Strengths and Limitations

A strength of this study is the use of a national quality registry that includes a large sample size consisting of older adults from different types of care units in urban and rural areas. Therefore, the sample may be regarded as representative, which strengthens the generalisability. Risk assessments made using DFRI and MNA-SF include several known risk factors, thus providing satisfactory content validity (Polit & Beck, 2020; Rosendahl et al., 2003). However, a potential limitation with DFRI is its reliance on the use of self-reported incidents of previous falls, which could result in the under- or overrepresentation of such events (Edvinsson et al., 2015). Another limitation is the need to update the instruments to better match the older population of today. The registry provides instructions to guide care staff in the risk assessments, which strengthens its reliability, but a limitation is non-adherence to the instructions, which can result in incorrect risk assessments and missing data. Care staff are challenged by high workloads and lack of time, conditions that might negatively impact the completeness and quality of data. Another important aspect is the competence of care staff, which may vary considerably. To correctly assess and decide upon which interventions to plan and perform requires professional skill and competence. The missing data for performed interventions in the present study may reflect shortcomings in the professional competence of care staff. It therefore may be necessary to include other professionals, such as physiotherapists, occupational therapists, dietitians, and dental health professionals, as part of the care team to improve the quality of care (Meranius & Josefsson, 2018). An additional limitation is that the data from quality registries like SA do not contain any medical or demographic data of the study participants; such data can be of importance in interpreting the results.

### Implications for Practice

The prevalence of negative events, such as malnutrition and falls, is increased among these older adults, and the consequences are extensive. These events are preventable, and a systematic, individualized risk assessment is a prerequisite for adequate interventions. A significant positive correlation between the risk of falling and the risk of malnutrition points towards the importance of a having a comprehensive approach to the prevention of falling and malnutrition. A low ratio between planned and performed interventions and sub-optimal adherence to guidelines suggests the need for supportive leadership, of implementation of clinical practice guidelines and education for care staff.

## Conclusions

This cross-sectional registry study showed a significant positive correlation between the risk of falling and the risk of malnutrition, which points towards the importance of a comprehensive assessment in the prevention of falling and malnutrition. It highlights the importance of using an evidence-based approach, based on adherence to recommended guidelines. Further, the implementation of clinical practice guidelines is needed, which requires educational training for care staff and supportive leadership. This study contributes to the body of knowledge on the risks of falling and malnutrition and highlights the overlooked actions in the prevention of falls and malnutrition in older adults living in municipal residential care in Sweden.
